# Design, Synthesis, and Cytotoxicity of 5-Fluoro-2-methyl-6-(4-aryl-piperazin-1-yl) Benzoxazoles

**DOI:** 10.3390/molecules21101290

**Published:** 2016-09-27

**Authors:** Thuraya Al-Harthy, Wajdi Michel Zoghaib, Maren Pflüger, Miriam Schöpel, Kamil Önder, Maria Reitsammer, Harald Hundsberger, Raphael Stoll, Raid Abdel-Jalil

**Affiliations:** 1 Chemistry Department, College of Science, Sultan Qaboos University, Muscat 123, Oman; thurayalharthi@gmail.com (T.A.-H.); zoghaibw@squ.edu.om (W.M.Z.); 2 IMC Fachhochschule Krems University of Applied Sciences Krems, Piaristengasse 1, Krems A-3500, Austria; maren.pflueger@fh-krems.ac.at (M.P.); harald.hundsberger@fh-krems.ac.at (H.H.); 3 Biomolecular NMR, Ruhr University of Bochum, Bochum 44780, Germany; miriam.schoepel@ruhr-uni-bochum.de (M.S.); raphael.stoll@ruhr-uni-bochum.de (R.S.); 4 Research Program for Rational Drug Design in Dermatology and Rheumatolog, Department of Dermatology, General Hospital of Salzburg, Paracelsus Medical, University of Salzburg, Müllner Hauptstraße 48, Salzburg A-5020, Austria; oender@procomcure.com (K.Ö.); m.reitsammer@salk.at (M.R.)

**Keywords:** benzoxazole, piperazine, fluorine Cytotoxicity, A-549 lung carcinoma cells, HepaRG hepatocytes

## Abstract

To design new compounds suitable as starting points for anticancer drug development, we have synthesized a novel series of benzoxazoles with pharmaceutically advantageous piperazine and fluorine moieties attached to them. The newly synthesized benzoxazoles and their corresponding precursors were evaluated for cytotoxicity on human A-549 lung carcinoma cells and non-cancer HepaRG hepatocyes. Some of these new benzoxazoles show potential anticancer activity, while two of the intermediates show lung cancer selective properties at low concentrations where healthy cells are unaffected, indicating a selectivity window for anticancer compounds.

## 1. Introduction

Despite all the progress in biomedical research, cancer remains a leading cause of death worldwide. Therefore, many efforts have been made to find new therapeutic agents that could potentially improve cancer therapy by using more selective and less toxic drugs.

Heterocyclic compounds are considered to be the most interesting drug-based structures in medicinal chemistry due to their widespread presence in lead compounds. Specifically, benzoxazoles have received much attention in recent years because of their broad spectrum of biological activities. Recent studies show that several benzoxazole analogues exhibit a number of biological activities including anticancer [1], antifungal [2], antituberculosis [3], mPGES-1 inhibitor [4], 5-HT receptor antagonist [5], CETP inhibitor [6], and antiplasmodial [7] activities.

Broad therapeutic spectrum compounds intrinsically possess a certain amount of cytotoxicity. Structural modification on lead anticancer compounds may eliminate or reduce cytotoxicity to a minimum level. Usually, a plethora of analogues are synthesized to decipher the underlying structure activity relationship (SAR) and to identify more selective, less toxic, and ADME-improved leads. Often, substitutions found to be bioactively advantageous on a certain active scaffold can be introduced to other scaffolds leading to the enhancement of bioactivity [8,9]. Fluorine and piperazine are common appendages and substituents in medicinal chemistry due to their immense utilities in drug design and their unique bioactivities [10,11].

**Figure 1:**
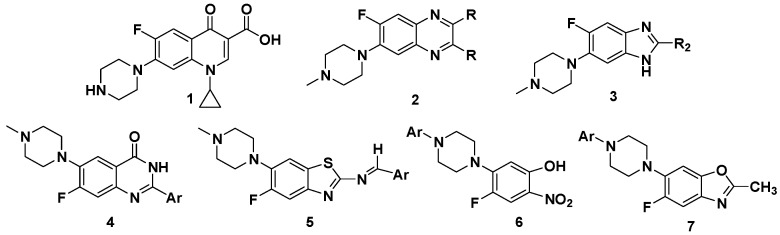
Examples of heterocyclic systems that contain fluorine and piperazine.

The introduction of fluorine and piperazine appendages played a vital role in enhancing the antimicrobial activities of the quinolone drugs, e.g., ciprofloxacin^®^ (**1**) (Figure 1), the most active antibiotic drug on the market [12]. Similarly, we have successfully synthesized a variety of heterocyclic systems incorporating fluorine and piperazine moieties, such as quinoxaline [13] (**2**), benzimidazole [14] (**3**), quinazolinone [15] (**4**), and recently benzothiazole [16] (**5**). Some derivatives of our systems showed very promising biological activities, which were attributed to the presence of both appendages. In this study, we combined the benzoxazole scaffold with both piperazine and fluorine moieties and evaluated their potential in anticancer activity.

Based on that, and as a part of our ongoing research in developing new potentially bioactive heterocyclic compounds, this study describes the synthesis of new 4-fluoro-5-(substituted phenyl-piperazin-1-yl)-2-nitro-phenoles (**6**) and their corresponding 5-fluoro-6-(substituted phenyl-piperazin-1-yl)-benzoxazoles (**7**) functionalized with both piperazine and fluorine moieties.

## 2. Results and Discussion

### 2.1. Chemistry

**Scheme 1:**
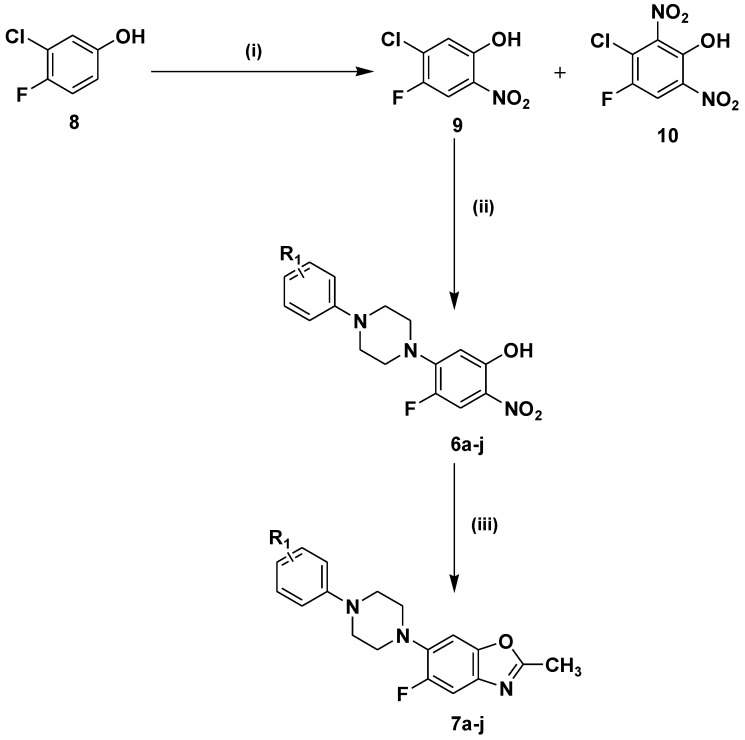
Synthesis of 5-fluoro-6-(substituted phenyl-piperazin-1-yl)-benzoxazoles; reagents and conditions: (i) 15% HNO_3_/AcOH, 0 °C, (ii) aryl piperazine, refluxing toluene or chlorobenzene (iii) In AcOH, benzene, trimethyl orthoacetate.

A new series of benzoxazole derivatives was synthesized using a multi-step protocol that involved regioselective nitration and piperazinylation, followed by a one-pot in-situ reductive cyclization step as depicted in Scheme 1.

To start with, the commercially available 3-chloro-4-fluorophenol (**8**) was nitrated using mild nitration conditions. The choice of a mild nitration step was effective and led to a shorter synthetic procedure compared to the use of strong acidic conditions. In fact, nitration yielded 5-chloro-4-fluoro-2-nitrophenol (**9**) and its 3-chloro-4-fluoro-2,6-dintirophenol (**10**) counterpart. Carefully controlling the reaction temperature, we were able to isolate **9** in a 60% yield after purification using flash column chromatography.

Subsequently, the piperazinylation of **9** using excess substituted phenyl *N*-piperazine yielded the key intermediates **6a**–**j**. Changing the solvent from dimethylsulfoxide to toluene simplified the work and considerably improved the yield up to 83% for **6a**, **6b**, **6d**, **6e**, **6g**, **6i**, and **6j** as shown in Table 1. For the preparation of **6c**, **6f**, and **6h** chlorobenzene was the solvent of choice, due to poor solubility of the corresponding aryl-piperazines in toluene nonetheless using chlorobenzene required a longer reaction time. Purification using column chromatography was required to remove unreacted *N*-piperazines.

The cyclization of intermediates (**6a**–**j**) was achieved by an indium-mediated reaction using indium/acetic acid followed by addition of trimethyl orthoaceate to form the corresponding benzoxazole derivatives (**7a**–**j**). This one-pot, reductive cyclization simplified and shortened the synthetic procedure and provided yields ranging from good to very good (53%–75%) after recrystallization as shown in Table 1. All the newly synthesized compounds were characterized using spectroscopic techniques. High-resolution mass spectrometry shows the correct molecular ion peaks. The formation of 3-chloro-4-fluoro-2,6-dinitrophenol (**10**) as a byproduct during the nitration step simultaneously with **9** was confirmed by ^1^H-NMR in which one and two types of ^1^H signals were evident in the spectra of **10** and **9**, respectively. In the ^1^H-NMR spectra of compounds (**7a**–**j**), the methyl proton signals appeared as singlets around δ 2.54–2.60 ppm. The H-4 protons were the most deshielded protons, showing a large ortho coupling with the adjacent fluorine *J*_H-F_ = 11.2–12.0 Hz compared with H-7 meta coupling *J*_H-F_ = 5.0–8.0 Hz. Piperazinyl protons resonated as broad singlets around δ 3.10–3.36 ppm. The disappearance of the exchangeable signals in intermediates (**6a**–**j**) around δ 10.40–10.80 ppm confirmed the successful cyclization to benzoxazoles. In ^13^C-NMR spectra of compounds (**7a**–**j**), the most deshielded carbon was C-2, resonating around δ 163.0–164.6 ppm. The methyl carbons appear in the upfield region around δ 13.5–14.6 ppm and the piperazine methylene carbons resonated around δ 48.0–52.0 ppm.

### 2.2. Cytotoxicity

The cytotoxicity of prepared benzoxazoles (**7a**–**j**) and intermediates (**6a**–**j** and **9**) were measured by applying the CellTiter-Glo^®^ luminescent cell proliferation assay (G755A, Promega, Madison, WI, USA). This method permits the determination of viable cells numbers in the culture based on quantitation of the ATP present, which denotes the presence of metabolically active cells [17]. In vitro cytotoxicity analysis was overall performed following the protocol as described in “Guidance Document on Using in Vitro Data to Estimate in Vivo Starting Doses for Acute Toxicity Based on Recommendations” [18]. Human A-549 cells are human lung cancer epithelial cells, which were chosen as the tumor model, whereas the HepaRG™ cell line is a classic hepatic cell line that retains many characteristics of primary human hepatocytes. HepaRG™ cells are terminally differentiated. In order to obtain CT_50_ values and detect a dose-dependent impact for each of the twelve test compounds, concentrations of 0, 0.1, 1.0, 10, 50, 100, 250, and 500 µM were analyzed with both cell lines, and doxorubicine (IC_50_ = 5.1 μM for HepaRG™ cells) was used as standard.

As shown in Table 2, we observed visible solubility problems for some compounds at concentrations of 50 µM, 100 µM, and 250 µM, while **6d** and **7f** were completely soluble at all concentrations. Therefore, a CT_50_ value of ~50 µM could only be obtained for **6d**, which shows equal toxicity to both cell lines, indicating a non-differential behavior.

Since the majority of test compound solutions with concentrations above 50 µM precipitated in cell-culture media, we performed cytotoxicity assays for healthy and cancer cells at 50 µM. For compounds **6i**, **7a**, **7d**, **7g**, **7h**, **7i**, and **7j**, it was not possible to draw any conclusion because of the precipitation problem.

Compared to healthy control cells, compounds **6a**, **6b**, **6g**, and **6j** showed significant increased toxicity in lung cancer A-549 cells >30%–40%. Interestingly, two compounds were more toxic to healthy cells compared with lung cancer cells **7b** and **7j**.

Even at concentrations as low as 10 µM, **6a** and **6g** killed ~40% of lung cancer cells, whereas all compounds were non-toxic to healthy cells. At a 10-µM concentration, nearly none of the tested compounds was toxic to healthy hepatocytes, but at least four compounds (**6a**, **6b**, **6g**, and **6j**) were highly toxic to lung cancer cells, killing 30%–40% of all cancer cells at this low concentration. Concentrations below 10 µM were not toxic at all, indicating a small activity window, which has to be further extended through improving efficacy in order to obtain useful compounds.

## 3. Materials and Methods

### 3.1. Chemistry

Reagents were obtained from commercial sources and used without further purification. All solvents were purchased from Sigma Aldrich (Munich, Germany) except for benzene and chlorobenzene, which were purchased from BDH. The staring materials, 3-chloro-4-fluorophenol and trimethyl orthoacetate, were purchased from Alfa Aesar (Munich, Germany).

The newly synthesized compounds were characterized using IR, ^1^H-NMR, ^13^C-NMR, HRMS, and a melting point. IR spectra (4000–400 cm^−1^) were recorded as KBr discs on a Perkin–Elmer Spectrum BX FTIR spectrophotometer (Akron, OH, USA). The ^1^H-NMR spectra were measured on Varian (Palo Alto, CA, USA) 400 MHz, and ^13^C-NMR spectra were recorded at 100 MHz using TMS as an internal standard assigned as zero ppm, and chemical shifts δ were given in part per million (ppm) downfield from TMS. The EI and FAB high-resolution mass spectra were recorded on a Finnigan MAT 312 mass spectrometer (ThermoFisher Scientific, Waltham, MA, USA) Melting points were determined on an SMP 10 Stuart Scientific apparatus (Keison Products, Essex, UK). The reactions were monitored by thin layer chromatography (TLC) using silica gel plates, and the components were visualized by UV light. Flash column chromatography was carried out using silica gel 60 (70–230 mesh) (Aldrich, Munich, Germany) as the stationary phase.

5-chloro-4-fluoro-2-nitrophenol (**9**) and 3-chloro-4-fluoro-2,6-dintirophenol (**10**): for the nitrating solution, 15% HNO_3_/AcOH was prepared and kept at 0 °C. 3-chloro-4-fluorophenol (**8**) (5.0 g, 34 mmol) was added in small portions to the solution and stirred for 1 h at 0 °C. The reaction mixture poured into an ice-water bath, filtered by suction, washed thoroughly with water, and then dried and purified by column chromatography 10% MeOH/CH_2_Cl_2_.

*5-Chloro-4-fluoro-2-nitro-phenol* (**9**) (yield 60%). m.p.: 82–83 °C. FTIR (KBr): 3471, 1629, 1530, 1330, 1247, 1150 cm^−1^. ^1^H-NMR (400 MHz, CDCl_3_) δ (ppm): 10.40 (bs, 1H, O-H), 7.91 (d, *J* = 8.4 Hz, H-6), 7.26 (d, *J* = 12.0 Hz, H-3). ^13^C-NMR (100 MHz, CDCl_3_) δ (ppm): 152.2(C), 151.7 (C), 150.0 (C), 132.5 (C), 121.8 (CH), 112.1 (CH). MS (ESI) [M] *m*/*z* calcd for C_6_H_3_ClFNO_3_: 190.98; found: 189.75.

*3-Chloro-4-fluoro-2,6-dintirophenol* (**10**) (yield 20%). ^1^H-NMR (400 MHz, CDCl_3_) δ (ppm): 10.60 (bs, 1H, O-H), 8.11 (d, *J* = 9.2 Hz, H-3). MS (ESI) [M] *m*/*z* calcd for C_6_H_2_ClFN_2_O_5_: 235.96; found: 234.79.

#### 3.1.1. General Procedure for Synthesis of 4-Fluoro-5-(substitutedphenyl-piperazin-1-yl)-2-nitro-phenol (**6a**, **6b**, **6d**, **6e**, **6g**, **6i**, **6j**)

*5-Chloro-4-fluoro-2-nitro-phenol* (**9**) (0.500 g, 2.61 mmol) was dissolved in toluene (5 mL), and the corresponding aryl piperazine (13.1 mmol) was then added and refluxed for 2–6 h. Afterwards, water was added and extracted with dichloromethane (3 × 30 mL), and the organic layer was concentrated, dried over sodium sulfate, and purified by flash column chromatography.

*4-Fluoro-2-nitro-5-(4-phenyl-piperazin-1-yl)-phenol* (**6a**) (yield 72%, 40% hexane/CH_2_Cl_2_). m.p.: 197–198 °C. FTIR (KBr): 3377, 1634, 1508, 1388, 1225 cm^−1^. ^1^H-NMR (400 MHz, CDCl_3_) δ (ppm): 10.90 (bs, 1H, O-H), 7.74 (d, *J* = 13.2 Hz, 1H, H-3), 6.46 (d, *J* = 7.6 Hz, 1H, H-6), 3.52 (s, 2H, piperazine), 3.34 (s, 2H, piperazine). ^13^C-NMR (100 MHz, CDCl_3_) δ (ppm): 153.9 (C), 150.6 (C), 148.3 (C), 145.9 (C), 129.3 (CH), 120.7 (C), 116.5 (CH), 112.0 (CH), 111.8 (CH), 105.2 (CH), 49.31 (CH_2_), 49.26 (CH_2_). HRMS (EI) *m*/*z* calcd for C_16_H_16_FN_3_O_3_: 317.1176; found: 317.1163.

*4-Fluoro-2-nitro-5-(4-p-tolyl-piperazin-1-yl)-phenol* (**6b**) (yield 73%, CH_2_Cl_2_). m.p.: 178–179 °C. FTIR (KBr): 3354, 1631, 1511, 1390, 1220 cm^−1^. ^1^H-NMR (400 MHz, CDCl_3_) δ (ppm): 10.84 (bs, 1H, O-H), 7.68 (d, *J* = 13.2 Hz, 1H, H-3), 6.83 (d, *J* = 7.2 Hz, 1H, H-6), 3.46 (s, 2H, piperazine), 3.22 (s, 2H, piperazine), 2.23 (s, 1H, CH_3_-Ph). ^13^C-NMR (100 MHz, CDCl_3_) δ (ppm): 154.0 (C), 148.4 (C), 148.3 (C), 145.9 (C), 129.9 (CH), 124.8 (C), 124.7 (C), 117.0 (CH), 112.0 (CH), 105.3 (CH), 49.8 (CH_2_), 49.2 (CH_2_), 20.5 (CH_3_). HRMS (ESI) [M + H^+^] *m*/*z* calcd for C_17_H_18_FN_3_O_3_: 332.1405; found: 332.1407.

*4-Fluoro-5-[4-(4-fluoro-phenyl)-piperazin-1-yl]-2-nitro-phenol* (**6d**) (yield 73%, CH_2_Cl_2_). m.p.: 151–152 °C. FTIR (KBr): 3309, 3103, 1637, 1535, 1244 cm^−1^. ^1^H-NMR (400 MHz, CDCl_3_) δ (ppm): 10.88 (bs, 1H, O-H), 7.74 (d, *J* = 13.6 Hz, 1H, H-3), 6.47 (d, *J* = 7.60 Hz, 1H, H-6), 3.51 (s, 2H, piperazine), 3.25 (s, 2H, piperazine). ^13^C-NMR (100 MHz CDCl3) δ (ppm): 158.9 (C), 156.5 (C), 153.9 (C), 148.3 (C), 146.0 (C), 124.9 (C), 118.5 (CH), 115.7 (CH), 111.8 (CH), 105.3 (CH), 50.2 (CH_2_), 49.3 (CH_2_). HRMS (EI) *m*/*z* calcd for C_16_H_15_F_2_N_3_O_3_: 335.1081; found: 335.1066.

*4-Fluoro-5-[4-(4-methoxy-phenyl)-piperazin-1-yl]-2-nitro-phenol* (**6e**) (yield 27%, CH_2_Cl_2_). m.p.: 202–204 °C. FTIR (KBr): 3448, 2933, 1638, 1515, 1225 cm^−1^. ^1^H-NMR (400 MHz, CDCl_3_) δ (ppm): 10.84 (bs, 1H, O-H), 7.68 (d, *J* = 13.2 Hz, 1H, H-3), 6.41 (d, *J* = 7.6 Hz, 1H, H-6), 3.72 (s, 1H, OCH_3_), 3.47 (s, 2H, piperazine), 3.17 (s, 2H, piperazine). ^13^C-NMR (100 MHz, CDCl_3_) δ (ppm): 153.9 (C), 148.3 (C), 145.9 (C), 122.5 (C), 121.4 (C), 119.2 (C), 114.7 (CH), 112.0 (CH), 111.8 (CH), 105.5 (CH), 55.6 (CH_3_), 51.4 (CH_2_), 49.0 (CH_2_). HRMS (EI) *m*/*z* calcd for C_17_H_18_FN_3_O_4_: 347.1281; found: 347.1264.

*4-Fluoro-5-[4-(2-fluoro-phenyl)-piperazin-1-yl]-2-nitro-phenol* (**6g**) (yield 83%, CH_2_Cl_2_). m.p.: 180–181 °C. FTIR (KBr): 3457, 2928, 1636, 1508, 1265 cm^−1^. ^1^H-NMR (400 MHz, CDCl_3_) δ (ppm): 10.82 (bs, 1H, O-H), 7.69 (d, *J* = 13.2 Hz, 1H, H-3), 6.42 (d, *J* = 7.6 Hz, 1H, H-6), 3.51 (s, 2H, piperazine), 3.25 (s, 2H, piperazine). ^13^C-NMR (100 MHz, CDCl_3_) δ (ppm): 157.0 (C), 154.5 (C), 154.0 (C), 148.5 (C), 145.9 (C), 139.3 (C), 124.6 (CH), 123.3 (CH), 119.1 (CH), 116.2 (CH), 111.8 (CH), 105.4 (CH), 50.2 (CH_2_), 49.5 (CH_2_). HRMS (EI) *m*/*z* calcd for C_16_H_15_F_2_N_3_O_3_: 335.1081; found: 335.1070.

*4-Fluoro-5-[4-(3-methoxy-phenyl)-piperazin-1-yl]-2-nitro-phenol* (**6i**) (yield 73%, CH_2_Cl_2_). m.p.: 148–149 °C. FTIR (KBr): 3426, 2937, 1629, 1504, 1209 cm^−1^. ^1^H-NMR (400 MHz, CDCl_3_) δ (ppm): 10.83 (bs, 1H, O-H), 7.68 (d, *J* = 13.6 Hz, 1H, H-3), 6.52 (d, *J* =8.0 Hz, 1H, H-6), 3.74 (s, 1H, OCH_3_), 3.45 (s, 2H, piperazine), 3.28 (s, 2H, piperazine). ^13^C-NMR (100 MHz, CDCl_3_) δ (ppm): 160.6 (C), 153.9 (C), 151.9 (C), 148.3 (C), 145.9 (C), 130.0 (CH), 124.8 (C), 112.0 (CH), 111.8 (CH), 109.1 (CH), 105.2 (CH), 103.0 (CH), 55.2 (CH_3_), 49.2 (CH_2_), 49.1 (CH_2_). HRMS (EI) *m*/*z* calcd for C_17_H_18_FN_3_O_4_: 347.1281; found: 347.1297.

*5-[4-(3,4-Dichloro-phenyl)-piperazin-1-yl]-4-fluoro-2-nitro-phenol* (**6j**) (yield 33%, CH_2_Cl_2_). m.p.: 211–213 °C. FTIR (KBr): 3448, 2924, 1634, 1513, 1207 cm^−1^. ^1^H-NMR (400 MHz, CDCl_3_) δ (ppm): 10.80 (bs, 1H, O-H), 7.70 (d, *J* = 13.6 Hz, 1H, H-3), 6.42 (d, *J* = 7.6 Hz, 1H, H-6), 3.49 (s, 2H, piperazine), 3.30 (s, 2H, piperazine). ^13^C-NMR (100 MHz, CDCl_3_) δ (ppm): 153.9 (C), 149.8 (C), 148.3 (C), 148.0 (C), 145.9 (C), 133.0 (C), 130.7 (CH), 120.1 (C), 117.9 (CH), 115.9 (CH), 112.1 (CH), 105.4 (CH), 49.0 (CH_2_), 48.8 (CH_2_). HRMS (EI) *m*/*z* calcd for C_16_H_14_C_l2_FN_3_O_3_: 385.0396; found: 385.0374.

#### 3.1.2. General Procedure for Synthesis 5-[4-(*o*/*m*/*p*-Hydroxy-phenyl)-piperazin-1-yl]-4-fluoro-2-nitro-phenol (**6c**, **6f**, **6h**)

5-Chloro-4-fluoro-2-nitro-phenol (**9**) (0.250 g, 1.31 mmol) was dissolved in chlorobenzene (5 mL), substituted phenyl-piperazine (6.53 mmol) was added and refluxed overnight. The reaction mixture was extracted with water and dichloromethane, and the organic layer was concentrated and purified by flash column chromatography.

*4-Fluoro-5-[4-(4-hydroxy-phenyl)-piperazin-1-yl]-2-nitro-phenol* (**6c**) (yield 37%, 10% EtOAc/CH_2_Cl_2_). m.p.: 218–219 °C. FTIR (KBr): 3475, 3117, 1631, 1515, 1242, cm^−1^. ^1^H-NMR (400 MHz, CDCl_3_ with 2 drops of CD_3_OD) δ (ppm): 10.81 (bs, 1H, O-H), 7.67 (d, *J* = 13.2 Hz, 1H, H-3), 6.42 (d, *J* = 7.6 Hz, 1H, H-6), 3.46 (s, 2H, piperazine), 3.13 (s, 2H, piperazine). ^13^C-NMR (100 MHz, CDCl_3_ with 2 drops of CD_3_OD) δ (ppm): 152.8 (C), 147.5 (C), 147.4 (C), 147.3 (C), 144.9 (C), 123.8 (C), 118.5 (CH), 115.0 (CH), 110.7 (CH), 104.4 (CH), 50.4 (CH_2_), 50.4 (CH_2_). HRMS (EI) *m*/*z* calcd for C_16_H_16_FN_3_O_4_: 333.1125; found: 331.1141.

*4-Fluoro-5-[4-(2-hydroxy-phenyl)-piperazin-1-yl]-2-nitro-phenol* (**6f**) (yield 71%, CH_2_Cl_2_). m.p.: 176–178 °C. FTIR (KBr): 3452, 2923, 1636, 1520, 1227 cm^−1^. ^1^H-NMR (400 MHz, CDCl_3_) δ (ppm): 10.81 (bs, 1H, O-H), 7.70 (d, *J* = 13.2 Hz, 1H, H-3), 6.43 (d, *J* =8.0 Hz, 1H, H-6), 3.45 (s, 2H, piperazine), 2.99 (s, 2H, piperazine). ^13^C-NMR (100 MHz, DMSO) δ (ppm): 152.9 (C), 150.6 (C), 147.5 (C), 145.2 (C), 139.7 (C), 126.0 (C), 123.7 (CH), 116.1 (CH), 119.9 (CH), 119.3 (CH), 112.6 (CH), 106.5 (CH), 50.3 (CH_2_), 49.7 (CH_2_). HRMS (EI) *m*/*z* calcd for C_16_H_16_FN_3_O_4_: 333.1125; found: 333.1111.

*4-Fluoro-5-[4-(3-hydroxy-phenyl)-piperazin-1-yl]-2-nitro-phenol* (**6h**) (yield 28%, 25% EtOAc/CH_2_Cl_2_). m.p.: 207–208 °C. FTIR (KBr): 3448, 2924, 1636, 1506, 1260 cm^−1^. ^1^H-NMR (400 MHz, CDCl_3_) δ (ppm): 10.83 (bs, 1H, O-H), 7.69 (d, *J* = 13.2 Hz, 1H, H-3), 7.11 (m, 1H, H-6), 3.46 (s, 2H, piperazine), 3.28 (s, 2H, piperazine). ^13^C-NMR (100 MHz, DMSO) δ (ppm): 158.5 (C), 152.7 (C), 147.5 (C), 147.3 (C), 145.1 (C), 130.1 (CH), 126.1 (C), 112.9 (CH), 107.2 (CH), 107.0 (CH), 106.4 (CH), 103.0 (CH), 49.4 (CH_2_), 48.4 (CH_2_). HRMS (EI) *m*/*z* calcd for C_16_H_16_FN_3_O_4_: 333.1125; found: 333.1131.

#### 3.1.3. General Procedure for Synthesis of 5-Fluoro-6-(substitutedphenyl-piperazin-1-yl)-benzoxazoles (**7a**–**j**)

*Fluoro-5-(substituted phenyl-piperazin-1-yl)-2-nitro-phenol* (**6a**–**j**) (1 mmol) was added to a mixture of indium powder (8 mmol) and acetic acid (20 mmol) in benzene (2 mL). Trimethyl orthoacetate (8 mmol) in benzene (2 mL) was then added to the reaction mixture, stirred, and refluxed for 1–4 h. The reaction mixture was cooled to room temperature, washed with saturated sodium bicarbonate, extracted with dichloromethane (3 × 10 mL), and washed with water followed by saturated sodium chloride solution (brine). The organic extracts were dried over sodium sulfate anhydrous and concentrated using a rotary evaporator. The residue was then recrystallized from absolute ethanol to yield the corresponding benzoxazole.

*5-Fluoro-2-methyl-6-(4-phenylpiperazin-1-yl)-benzo[d]oxazole* (**7a**) (yield 71%). m.p.: 153–154 °C. FTIR (KBr): 2848, 1638, 1242 cm^−1^. ^1^H-NMR (400 MHz, CDCl_3_) δ (ppm): 7.27 (m, 1H, H-4), 7.06 (d, *J* = 6.8 Hz, 1H, H-7), 3.35 (s, 2H, piperazine), 3.22 (s, 2H, piperazine), 2.54 (s, 3H, CH_3_). ^13^C-NMR (100 MHz, CDCl_3_) δ (ppm): 163.1 (C), 153.9 (C), 151.5 (C), 150.0 (C), 137.2 (C), 134.9 (C), 128.2 (CH), 119.3 (CH), 115.4 (CH), 105.4 (CH), 99.5 (CH), 50.2 (CH_2_), 48.6 (CH_2_), 13.5 (CH_3_). HRMS (EI) *m*/*z* calcd for C_18_H_18_FN_3_O: 311.1434; found: 311.1423.

*5-Fluoro-2-methyl-6-(4-p-tolyl-piperazin-1-yl)-benzooxazole* (**7b**) (yield 53%). m.p.: 168–170 °C. FTIR (KBr) 1636, 1265 cm^−1^. ^1^H-NMR (400 MHz, CDCl_3_) δ (ppm): 7.27 (d, *J* =11.6 Hz, 1H, H-4), 7.06 (m, 1H, H-7), 3.28 (s, 2H, piperazine), 3.19 (s, 2H, piperazine), 2.54 (s, 3H, CH_3_), 2.23 (s, 3H, CH_3_-Ph). ^13^C-NMR (100 MHz, CDCl_3_) δ (ppm): 164.1 (C), 152.5 (C), 149.0 (C), 147.5 (C), 138.2 (C), 135.9 (C), 135.8 (C), 129.7 (CH), 116.8 (CH), 106.2 (CH), 100.5 (CH), 51.2 (CH_2_), 50.1(CH_2_), 20.5 (CH_3_), 14.6 (CH_3_). HRMS (EI) *m*/*z* calcd for C_19_H_20_FN_3_O: 325.1590; found: 325.1577.

*4-[4-(5-Fluoro-2-methyl-benzooxazol-6-yl)-piperazin-1-yl]-phenol* (**7c**) (yield 63%). m.p.: 188–191 °C. FTIR (KBr): 3466, 2937, 1640, 1267 cm^−1^. ^1^H-NMR (400 MHz, DMSO) δ (ppm): 8.90 (bs, 1H, O-H), 7.48 (d, *J* = 12.0 Hz, 1H, H-4), 7.38 (d, *J* = 7.2 Hz, 1H, H-7), 3.36 (s, 2H, piperazine), 3.10 (s, 2H, piperazine), 2.54 (s, 3H, CH_3_). ^13^C-NMR (100 MHz, DMSO) δ (ppm): 164.6 (C), 154.6 (C), 152.2 (C), 147.6 (C), 144.5 (C), 138.4 (C), 135.5 (C), 118.5 (CH), 115.9 (CH), 106.5 (CH), 101.6 (CH), 51.3 (CH_2_), 50.6 (CH_2_), 14.6 (CH_3_). HRMS (EI) *m*/*z* calcd for C_18_H_18_FN_3_O_2_: 327.1383; found: 327.1391.

*5-Fluoro-6-[4-(4-fluoro-phenyl)-piperazin-1-yl]-2-methyl-benzooxazole* (**7d**) (yield 51%). m.p.: 148–150 °C. FTIR (KBr): 2928, 1640, 1267 cm^−1^. ^1^H-NMR (400 MHz, CDCl_3_) δ (ppm): 7.27 (d, *J* = 11.6 Hz, 1H, H-4), 7.06 (d, *J* = 7.2 Hz, 1H, H-7), 3.20 (s, 2H, piperazine), 2.25 (s, 2H, piperazine), 2.54 (s, 3H, CH_3_). ^13^C-NMR (100 MHz, CDCl_3_) δ (ppm): 164.3 (C), 154.9 (C), 152.2 (C), 147.6 (C), 144.5 (C), 138.4 (C), 135.5 (C), 118.5 (CH), 115.9 (CH), 106.5 (CH), 101.6 (CH), 51.6 (CH_2_), 51.0 (CH_2_), 14.6 (CH_3_). HRMS (EI) *m*/*z* calcd for C_18_H_17_F_2_N_3_O: 329.1340; found: 329.1352.

*5-Fluoro-6-[4-(4-methoxy-phenyl)-piperazin-1-yl]-2-methyl-benzooxazole* (**7e**): (yield 70%). m.p.: 156–157 °C. FTIR (KBr): 2946, 1636, 1236 cm^−1^. ^1^H-NMR (400 MHz, CDCl_3_) δ (ppm): 7.27 (d, *J* = 11.6 Hz, 1H, H-4), 7.06 (d, *J* = 7.2 Hz, 1H, H-7), 3.72 (s, 3H, OCH_3_), 3.21 (s, 2H, piperazine), 3.20 (s, 2H, piperazine), 2.54 (s, 3H, CH_3_). ^13^C-NMR (100 MHz, CDCl_3_) δ (ppm): 164.3 (C), 153.9 (C), 151.5 (C), 146.5 (C), 144.2 (C), 137.2 (C), 134.8 (C), 117.6 (CH), 113.5 (CH), 105.2 (CH), 99.5 (CH), 54.5 (CH_3_), 50.2 (CH_2_), 50.0 (CH_2_), 13.5 (CH_3_). HRMS (EI) *m*/*z* calcd for C_19_H_20_FN_3_O_2_: 341.1540; found: 341.1548.

*2-[4-(5-Fluoro-2-methyl-benzooxazol-6-yl)-piperazin-1-yl]-phenol* (**7f**) (yield 55%). m.p.: 172–173 °C. FTIR (KBr): 3453, 2928, 1636, 1265 cm^−1^. ^1^H-NMR (400 MHz, CDCl3) δ (ppm): 7.28 (d, *J* = 11.2 Hz, 1H, H-4), 6.94 (d, *J* = 5.0 Hz, 1H, H-7), 3.24 (s, 2H, piperazine), 3.10 (s, 2H, piperazine), 2.55 (s, 3H, CH_3_). ^13^C-NMR (100 MHz, CDCl_3_) δ (ppm): 163.2 (C), 153.9 (C), 151.5 (C), 150.4 (C), 146.5 (C), 137.0 (C), 134.9 (C), 125.7 (CH), 120.5 (CH), 119.2 (CH), 113.2 (CH), 105.5 (CH), 99.6 (CH), 51.6 (CH_2_), 50.8 (CH_2_), 13.6 (CH_3_). HRMS (EI) *m*/*z* calcd for C_18_H_18_FN_3_O_2_: 327.1383; found: 327.1369.

*5-Fluoro-6-(4-(2-fluorophenyl)-piperazin-1-yl)-2-methylbenzo[d]oxazole* (**7g**) (yield 74%). m.p.: 157–158 °C. FTIR (KBr): 2834, 1634, 1271 cm^−1^. ^1^H-NMR (400 MHz, DMSO) δ (ppm): 7.49 (d, *J* = 12.0 Hz, 1H, H-4), 7.42 (d, *J* = 7.6 Hz, 1H, H-7), 3.36 (s, 2H, piperazine), 3.15 (s, 2H, piperazine), 2.54 (s, 3H, CH_3_). ^13^C-NMR (100 MHz, DMSO) δ (ppm): 164.7 (C), 156.7 (C), 154.4 (C), 147.6 (C), 140.1 (C), 138.4 (C), 135.7 (C), 125.4 (CH), 123.0 (CH), 119.8 (CH), 116.4 (CH), 106.4 (CH), 101.8 (CH), 51.3 (CH_2_), 50.6 (CH_2_), 14.6 (CH_3_). HRMS (EI) *m*/*z* calcd for C_18_H_17_F_2_N_3_O: 329.1340; found: 329.1353.

*3-[4-(5-Fluoro-2-methyl-benzooxazol-6-yl)-piperazin-1-yl]-phenol* (**7h**) (yield 74%). m.p.: 211–213 °C. FTIR (KBr): 3448, 2941, 1622, 1278 cm^−1^. ^1^H-NMR (400 MHz, CDCl_3_) δ (ppm): 7.28 (d, *J* = 11.6 Hz, 1H, H-4), 7.12 (d, *J* = 8.0 Hz, 1H, H-7), 3.35 (s, 2H, piperazine), 3.22 (s, 2H, piperazine), 2.55 (s, 3H, CH_3_). ^13^C-NMR (100 MHz, DMSO) δ (ppm): 164.7 (C), 158.5 (C), 154.6 (C), 152.7 (C), 147.6 (C), 138.4 (C), 135.6 (C), 130.1 (CH), 107.2 (CH), 106.9 (CH), 106.2 (CH), 103.0 (CH), 101.8 (CH), 51.1 (CH_2_), 48.9 (CH_2_), 14.5 (CH_3_). HRMS (EI) *m*/*z* calcd for C_18_H_18_FN_3_O_2_: 327.1383; found: 327.1368.

*5-Fluoro-6-[4-(3-methoxy-phenyl)-piperazin-1-yl]-2-methyl-benzooxazole* (**7i**) (yield 77%). m.p.: 124–125 °C. FTIR (KBr): 2942, 1636, 1280 cm^−1^. ^1^H-NMR (400 MHz, CDCl_3_) δ (ppm): 7.33 (d, *J* = 11.2 Hz, 1H, H-4), 7.11 (d, *J* = 7.2 Hz, 1H, H-7), 3.80 (s, 3H, OCH_3_), 3.38 (s, 2H, piperazine), 3.24 (s, 2H, piperazine), 2.60 (s, 3H, CH_3_). ^13^C-NMR (100 MHz, CDCl_3_) δ (ppm): 164.2 (C), 160.6 (C), 154.9 (C), 152.5 (C), 147.5 (C), 138.2 (C), 135.8 (C), 129.9 (CH), 109.1 (CH), 106.2 (CH), 104.8 (CH), 102.8 (CH), 100.5 (CH), 55.2 (CH_3_), 51.2 (CH_2_), 49.4 (CH_2_), 14.5 (CH_3_). HRMS (EI) *m*/*z* calcd for C_19_H_20_FN_3_O_2_: 341.1540; found: 341.1528.

*6-[4-(3,4-Dichloro-phenyl)-piperazin-1-yl]-5-fluoro-2-methyl-benzooxazole* (**7j**) (yield 75%). m.p.: 147–148 °C. FTIR (KBr): 2928, 1638, 1272 cm^−1^. ^1^H-NMR (400 MHz, CDCl_3_) δ (ppm): 7.28 (d, *J* = 11.6 Hz, 1H, H-4), 7.06 (d, *J* = 7.2 Hz, 1H, H-7), 3.31 (s, 2H, piperazine), 3.17 (s, 2H, piperazine), 2.55 (s, 3H, CH_3_). ^13^C-NMR (100 MHz, CDCl_3_) δ (ppm): 163.3 (C), 153.9 (C), 151.5 (C), 149.3 (C), 146.5 (C), 136.7 (C), 135.2 (C), 131.9 (C), 129.5 (CH), 116.7 (CH), 114.7 (CH), 105.3 (CH), 99.6 (CH), 49.8 (CH_2_), 48.1 (CH_2_), 13.6 (CH_3_). HRMS (ESI) [M + H^+^] *m*/*z* calcd for C_18_H_17_C_l2_FN_3_O: 380.0727; found: 380.0729.

### 3.2. Cytotoxicity

Human cell cultures: Hepatocytes (HepaRG^TM^ cells) were obtained from Life Technologies, and were cultivated in William’s E Medium (Gibco, Life Technologies, Waltham, MA, USA) and supplemented with 1x GlutaMAX™ plus 1x HepaRG^TM^ General Purpose Medium Supplement in 25-cm^2^ or 75-cm^2^ flasks (TPP, Trasadingen, Switzerland) at 37 °C and 5% CO_2_. Lung cancer epithelial cells A-549 were propagated in Epilife Medium (Gibco, Life Technologies; MEPICF) and combined with CaCl_2_ and 1x-growth-supplement containing penicillin and streptomycin (Gibco, Life Technologies). For sub-culturing, cells were split at a ratio of 1:4 to 1:8. For cryopreservation, cells were frozen in 10% DMSO in liquid nitrogen.

Human cytotoxicity assay: Human hepatocytes or human lung carcinoma cells were seeded into sterile black clear-bottomed polystyrene Corning^®^ CellBind^®^ 96-well plates (Munich, Germany) at a density of 20,000 cells/well after calculating cell numbers from trypsinized samples in a classical Neubauer Improved hemocytometer (VWR, Munich, Germany). The cells were grown for 24 h at 37 °C in a 5% CO_2_ atmosphere in Epilife medium (keratinocytes) or William′s E medium (hepatocytes). After a 24-h recovery period, media were removed from the wells by aspiration. The cells were then immediately exposed to the respective test compounds or reference antibiotics (prepared in advance in 100 µL of the same media) for 24 h 37 °C with 5% CO_2_. The concentration tested ranged from 0.1 µM to 500 µM (0.1 µM, 1 µM, 10 µM, 50 µM, 100 µM, 250 µM, and 500 µM) and was formulated as a 100× concentrated stock each, resulting in a final constant DMSO concentration of 1% per 100-µL sample volume. Test series were each performed in triplicate, and additionally included a vehicle reference (DMSO only) plus another internal control for evaluating overall assay performance. Additionally, cells were visually examined under a light microscope for any morphological abnormalities relative to the untreated cells. Subsequently, cell viability, as an indicator of potential cytotoxicity, was measured by applying the CellTiter-Glo^®^ luminescent cell proliferation assay (G755A, Promega, Madison, WI, USA). This method permits the determination of the number of viable cells in culture based on quantitation of the ATP present, which denotes the presence of metabolically active cells. To this end, 100 µL (equal to the volume of culture medium) of the kit-provided CellTiter-Glo^®^ reagent (reconstituted by transferring the thawed CellTiter-Glo^®^ buffer to the lyophilized enzyme and substrate mixture) was directly added to each well with a repetitive dispensing pipette. The contents were then mixed for 2 min at room temperature on an orbital minishaker (IKA^®^ MS3 digital, Staufen, Germany) at 450 rpm to induce efficient cell lysis, and then further incubated for 10 min at room temperature for stabilization of the resultant luminescent signal. Luminescence was recorded using a compatible multimode reader (GloMax^®^ Multi Detection Platform, Promega Corporation, Madison, WI, USA) with its embedded, preset CellTiter-Glo^®^ parameters (integration time of 0.5 s). Output values were expressed as percent cell viability compared with the vehicle (DMSO) control, and EC_50_ values were calculated from the dose-response curves via nonlinear regression (version 7, Graphpad Prism Software Inc., La Jolla, CA, USA).

## 4. Conclusions

A novel series of 5-fluoro-2-methyl-6-(4-arylpiperazin-1-yl)benzoxazoles (**7a**–**j**) incorporating both fluorine and piperazine were prepared in good yields. Initial cytotoxicity analysis of some of the intermediates—4-fluoro-5-(substituted phenylpiperazin-1-yl)-2-nitrophenols (**6a**–**j**)—showed promising activities and cell type-dependent cytotoxicity. Although the best activity achieved is still too low, the lung cancer sensitive pattern is a crucial property underlining a therapeutically interesting window, which can be further investigated. The poor solubility of some benzoxazoles could be improved by replacing the aryl-piperazine with *N*-methylpiperazine at position 6 and replacing the methyl moiety at position 2 with a carbamate functional group. Manipulating the benzoxazole structure is now under intensive investigation in our laboratory, and the results will be published in due course. Finally, at least two compounds (**6d** and **6g**) can be used as starting points for the development of selective anti-cancer compounds in the future.

## Figures and Tables

**Table 1:** Data for the synthesis of benzoxazoles and their precursors.

Compound	R	Time (h)	Yield * %	Solvent **	Compound	Time (h)	Yield * %	Solvent **
**6a**	H	5.5	72	Toluene	**7a**	4	71	Benzene
**6b**	*p*-CH_3_	2	73	Toluene	**7b**	3	53	Benzene
**6c**	*p*-OH	24	37	Chlorobenzene	**7c**	4	63	Benzene
**6d**	*p*-F	2	73	Toluene	**7d**	2.5	51	Benzene
**6e**	*p*-OCH_3_	1.5	27	Toluene	**7e**	1	70	Benzene
**6f**	*o*-OH	3	71	Chlorobenzene	**7f**	1.5	55	Benzene
**6g**	*o*-F	2.5	83	Toluene	**7g**	2	74	Benzene
**6h**	*m*-OH	24	28	Chlorobenzene	**7h**	2	74	Benzene
**6i**	*m*-OCH_3_	2	73	Toluene	**7i**	1	77	Benzene
**6j**	3,4-Cl	5	33	Toluene	**7j**	1	75	Benzene

* The yield was calculated after purification by crystallization or column chromatography. ** Solvent at reflux temperature.

**Table 2:** Cell viability shown in % for benzoxazole derivatives and their intermediates.

Concentration (C) µM																
Liver Cells, Hepatocytes									Lung Cancer Cells							
Compound	0	0.1	1	10	50	100	250	500	0	0.1	1	10	50	100	250	500
**6a**	100	86	97	112	90	76			100	97	104	64	61	58		
**6b**	100	107	123	120	97	96			100	99	100	68	65			
**6c**	100	95	101	110	101	89			100	104	103	102	94			
**6d**	100	94	87	101	57	62	44	13	100	103	111	78	58	54	46	34
**6e**	100	107	110	104	74	79			100	101	104	96	71			
**6f**	100	105	104	110	76				100	97	100	93	60	46		
**6g**	100	110	109	112	87				100	96	96	62	51	42		
**6h**	100	117	109	108	83	58			100	106	106	105	87			
**6i**	100	79	95	99					100	95	93	96	69			
**6j**	100	119	115	81	102	108			100	98	94	65	62			
**7a**	100	101	97	94					100	101	96	90	60			
**7b**	100	105	99	91	63	60			100	92	94	92	87	75		
**7c**	100	98	107	101	93				100	92	97	101	90	83		
**7d**	100	91	94	85					100	98	98	87	87	82		
**7e**	100	107	108	91	71				100	93	92	75	74	68		
**7f**	100	106	97	86	59	60	53	54	100	103	99	91	91	95	83	63
**7g**	100	96	106	87					100	96	92	92	73	63		
**7h**	100	100	102	99					100	100	93	97	95	90		
**7i**	100	100	102	91					100	105	102	88	76	75		
**7j**	100	100	90	85	65	27	7	7	100	98	94	81	70	47	28	
